# Topical Insulin Application in the Management of Resistant Leg Ulcers in a Patient With Prolidase Deficiency: A Case Report

**DOI:** 10.7759/cureus.47672

**Published:** 2023-10-25

**Authors:** Yusuf Can Edek, Hale Nur Ertugay Aral, Esra Adışen, Ahmet Burhan Aksakal

**Affiliations:** 1 Dermatology, Gazi University, Ankara, TUR

**Keywords:** topical treatment, treatment, prolidase deficiency, leg ulcer, insulin

## Abstract

Leg ulcers are a significant cause of morbidity and mortality and can be caused by vascular, neuropathic, infectious, and traumatic factors, as well as rare metabolic diseases like prolidase deficiency. Despite various wound care methods and systemic treatments, managing ulcers can be challenging.

This case presents a male patient with prolidase deficiency for 35 years whose leg ulcers were resistant to standard treatments such as wound dressings, topical treatments, and hyperbaric oxygen therapy. Considering the ulcers' resistant nature, we applied topical insulin to ulcers as an add-on therapy and observed clinical improvement. In this case, we want to emphasize the potential of insulin as a supplementary treatment agent in prolidase deficiency-induced ulcer treatment.

## Introduction

The etiological agents of leg ulcers can be diverse, stemming from vascular, neuropathic, infectious, and traumatic factors. Moreover, rare metabolic diseases, such as prolidase deficiency, can also be the culprits behind leg ulcers [1,2].

Prolidase deficiency is an autosomal recessive genodermatosis manifested by chronic ulcers, distinctive facial appearance, mental retardation, and recurrent infections. This deficiency is due to an absence of the prolidase enzyme, crucial in the metabolism of iminodipeptides such as proline and hydroxyproline. Elevated circulating levels of proline and hydroxyproline can impair collagen synthesis. The prolidase enzyme is also linked to inflammatory and angiogenic signaling pathways; thus, its deficiency is associated with delayed wound healing [2-5].

The disease's characteristic dermatological manifestation includes treatment-resistant ulcers that can scar, accompanied by granulation tissue and purulent exudate with irregular boundaries. Other findings can include telangiectasias, purpura, premature graying of hair, photosensitivity, itching, lymphedema, and hyperkeratosis in the knees and elbows.

While enzyme replacement therapies are under exploration, there's no definitive treatment for the disease. The focus mainly lies on managing dermatological symptoms. Various topical, systemic, and surgical treatments are utilized in ulcer management. Ulcers in prolidase deficiency typically prove resistant to standard wound care methods. In literature, cases treated with dapsone, systemic steroids, pentoxifylline, oral proline, ascorbic acid, topical glycine, proline, erythrocyte apheresis, and skin grafting have been reported. Despite trying various treatment options, the treatment response rates are generally not high. This situation underscores the need for exploring new treatment modalities [2-5].

In this case presentation, we aim to introduce a male patient diagnosed with prolidase deficiency whose leg ulcers proved resistant to standard treatments. Consequently, topical insulin was employed for ulcer management. With this case, we emphasize the potential of insulin, data on whose application in ulcer management is steadily growing, as a supplementary treatment agent in prolidase deficiency-induced ulcer treatment.

## Case presentation

**Figure 1:**
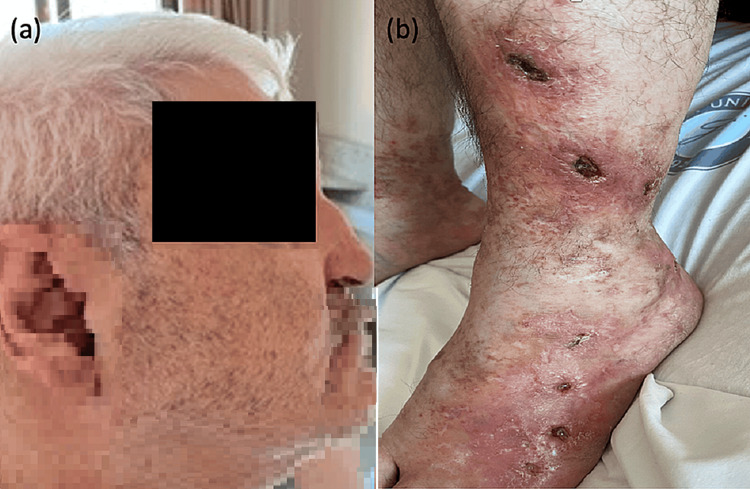
Patient photographs at the time of the presentation (a) Dysmorphic facial features - depressed nasal bridge, premature graying of hair, and telangiectasias on the cheeks, (b) two ulcers on the left leg measuring 1.5x1 cm and 1x1 cm, with ulcerative lesions on the dorsum of the left foot, the largest measuring 0.5x0.5 cm.

A 45-year-old male patient diagnosed with prolidase deficiency was evaluated by our team due to leg ulcers. The patient's history revealed recurrent ulcers for 35 years, accompanying mental retardation, and dysmorphic facial features (a depression at the nasal root, premature graying of hair, and telangiectasias on cheeks). About a decade ago, the erythrocyte prolidase enzyme level was identified to be reduced (0.16 μmol/pro/min/g Hb (Reference value: 2.69 μmol/pro/min/g Hb)), resulting in a diagnosis of prolidase deficiency. The patient, with a chronic trajectory of leg ulcers, had previously used wound dressings, various topical treatments, and hyperbaric oxygen therapy. Although he observed some benefits, he approached us due to a recent increase in the size and exudation of the ulcers. Dermatological examination revealed two ulcers on the left leg, measuring 1.5x1 cm and 1x1 cm respectively, and ulcers on the dorsum of the left foot, the largest being 0.5x0.5 cm in dimension (Figure 1).

**Figure 2:**
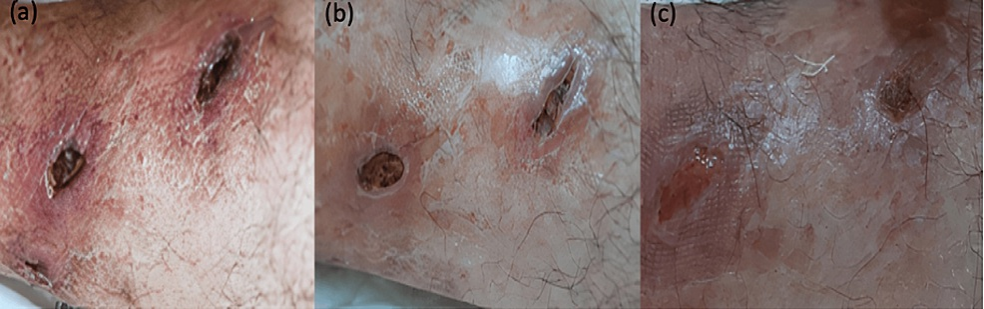
Ulcer photographs during the treatment Appearance of the ulcers on the left leg before topical insulin treatment (a), after the first week of treatment (b), and after the fourth week of treatment (c).

Histopathological analysis of skin biopsy from the edge of the ulcer demonstrated capillary proliferation, edema, and mixed-type inflammation in the dermis. A Doppler ultrasound of the lower extremities found no pathology, yet the culture from the ulcer displayed the growth of *Staphylococcus aureus* and *Pseudomonas aeruginosa*. Hence, intravenous ampicillin-sulbactam antibiotic therapy was administered for a fortnight. Following antibiotic treatment, a decrease in exudation was observed. The ulcers were topically treated with fusidic acid+betamethasone valerate cream twice daily, accompanied by local wound care. Considering the ulcers' resistant nature and literature suggesting the potential efficacy of topical insulin application, a decision was made to employ topical insulin on the patient's ulcers. Insulin treatment (Insulin aspart 100IU/ml®) was applied by dripping onto the ulcer edges using an insulin syringe twice daily, totaling 1 ml, and subsequently covered with sterile gauze. Both pre and post-treatment, the patient's blood sugar levels were within normal limits. By the first week of treatment, a reduction in ulcer size was evident, and by the end of the month, significant healing of the lesion was observed and compression therapy was suggested during follow-up with the patient (Figure 2).

## Discussion

Insulin is primarily recognized for its crucial roles in metabolism, such as the regulation of blood glucose and lipid levels. The potential effect of systemic insulin on wound healing has spurred the idea that it could serve as an affordable and effective agent for this purpose. However, the occurrence of side effects like hypoglycemia and hypokalemia in systemic application has limited its use. This led to the proposition of applying insulin topically to wounds to circumvent these side effects [6,7].

While the precise mechanism of action for topical insulin in treatment is not clearly understood, various pathways and molecules associated with its effects have been reported. In a randomized controlled study by Chen et al. to comprehend the effects of local insulin application on the wound healing process, one group was treated with topical insulin for ulcers while the other received saline [8]. At the end of the study, it was observed that topical insulin application to the ulcerative lesion stimulated macrophage infiltration in the area, increased MCP-1 expression and phagocytosis, and elevated the release of inflammatory cytokines. As insulin demonstrates these effects, it also induces an increase in M2-macrophage and interleukin (IL)-10 levels, triggering the migration of keratinocytes, fibroblasts, and endothelial cells to the wound site and playing a role in angiogenesis and granulation tissue formation. All of these contribute significantly to the wound-healing process. The epithelialization and wound maturation effects of insulin are effectively achieved by the PI3K-Akt-Rac1 signaling pathway, according to Liu et al. (2018) [9] and Liu et al. (2009) [10] studies.

There are case reports and publications on the use of topical insulin in many fields including dermatology, surgery, and ophthalmology. In dermatological literature, data exists on its application in diabetic ulcers, aphthous ulcers, pemphigus lesions, diabetic ulcers, and stasis ulcers, and successful treatment results were published in these cases [7,11-14].

## Conclusions

In our case, we observed a rapid reduction in ulcer size in the leg following the application of topical insulin. While numerous therapeutic agents exist for ulcer management in prolidase deficiency, we wish to highlight that topical insulin could be one of these methods through this case report. It presents as an affordable and effective agent in wound care.
